# Fecaloma-Associated Sigmoid Colon Perforation: A Case Report

**DOI:** 10.7759/cureus.102389

**Published:** 2026-01-27

**Authors:** Ahmed A Alharthi, Anas E Ahmed, Arafah A Alsayed, Naif A Alsokih, Husain A Alghanem

**Affiliations:** 1 College of Medicine, Universiti Sains Malaysia, Kubang Kerian, MYS; 2 Department of Community Medicine, Jazan University, Jazan, SAU; 3 College of Medicine, Umm Al-Qura University, Mecca, SAU; 4 College of Medicine, Hail University, Hail, SAU; 5 College of Medicine, King Faisal University, Al-Ahsa, SAU

**Keywords:** acute abdomen, bowel ischemia, chronic constipation, colonic perforation, elderly patient, exploratory laparotomy, fecaloma, hartmann’s procedure, sigmoid colon perforation, stercoral perforation

## Abstract

Fecaloma represents an uncommon yet potentially life-threatening consequence of long-standing constipation. It refers to a densely compacted fecal mass that may result in serious sequelae such as intestinal obstruction, compromised bowel perfusion, and perforation. We describe the case of a 68-year-old woman with known chronic constipation and chronic kidney disease who presented with sudden-onset abdominal pain, progressive distension, and clinical features suggestive of a systemic inflammatory response. Computed tomography (CT) demonstrated a sizable fecaloma within the sigmoid colon along with free intraperitoneal air, raising concern for colonic perforation. The patient underwent emergency exploratory laparotomy, which revealed a perforation of the sigmoid colon adjacent to the fecaloma, and a Hartmann’s procedure was subsequently performed. The postoperative recovery was uncomplicated, and the patient was discharged in good clinical condition. This report underscores the need to maintain a high index of suspicion for stercoral perforation in elderly patients with chronic constipation who present with an acute abdomen. Prompt diagnosis and timely surgical management are essential, as delayed treatment is linked to significant morbidity and mortality. Heightened clinical awareness of this rare but severe condition may contribute to improved patient outcomes.

## Introduction

Fecaloma is characterized by the formation of a dense, dehydrated mass of fecal matter that becomes firmly lodged within the colon and is considered an advanced manifestation of chronic constipation [1]. Although uncommon, it predominantly affects older individuals, as well as patients with neurological disorders, reduced mobility, or prolonged impairment of normal bowel motility and evacuation [2]. In contrast to uncomplicated fecal impaction, fecalomas are frequently resistant to conventional medical therapies and may give rise to severe complications such as intestinal obstruction, colonic ischemia, mucosal ulceration, and, in rare instances, bowel perforation. Perforation resulting from a fecaloma constitutes a medical and surgical emergency and carries a high risk of morbidity and mortality, particularly when recognition is delayed or the presentation is mistaken for more common etiologies of acute abdominal pain [1,2].

The underlying mechanism of colonic perforation is thought to involve sustained intraluminal pressure leading to ischemic necrosis of the bowel wall, most commonly occurring in the rectosigmoid region, where fecal contents tend to be most compacted [3-6]. Because early clinical manifestations are often subtle and nonspecific, diagnosis may be challenging. Early identification and prompt surgical management are therefore crucial for optimizing patient outcomes. In this report, we present a case of sigmoid colon perforation caused by a fecaloma in an elderly woman with a history of chronic constipation, with emphasis on the diagnostic process, therapeutic considerations, and broader clinical significance of this rare but potentially fatal condition.

## Case presentation

A 68-year-old woman with a medical background notable for long-standing constipation, hypertension, and stage II chronic kidney disease was brought to the emergency department due to a three-day course of escalating abdominal pain, increasing abdominal girth, and complete absence of bowel movements or flatus. She described a chronic history of infrequent, hard stools that frequently necessitated the use of stimulant laxatives and, at times, enemas. During the week preceding admission, she experienced reduced oral intake, nausea without emesis, and generalized lethargy. On the day of presentation, she developed abrupt-onset, severe, and diffuse abdominal pain that progressed rapidly and was associated with lightheadedness and transient confusion. She denied any recent abdominal trauma, prior surgical procedures, or known gastrointestinal malignancy.

On physical examination, the patient appeared toxic and uncomfortable, with mild diaphoresis. Vital signs were concerning for systemic illness, including a temperature of 38.3°C, hypotension (96/58 mmHg), tachycardia (112 beats per minute), tachypnea (24 breaths per minute), and an oxygen saturation of 95% while breathing ambient air. The abdomen was markedly distended and diffusely tender, with maximal discomfort noted in the lower quadrants. Signs of peritoneal irritation, including guarding and rebound tenderness, were present, and bowel sounds were diminished. Digital rectal examination identified a large, fixed, firm intraluminal mass consistent with fecal impaction, without gross blood.

Laboratory evaluation demonstrated significant leukocytosis, with a white blood cell count of 17.4 × 10⁹/L and neutrophil predominance (88%). Hemoglobin was measured at 12.8 g/dL, and inflammatory markers were elevated, with a C-reactive protein level of 86 mg/L. Renal function was mildly impaired compared to baseline, with a serum creatinine of 2.1 mg/dL (baseline: 1.6 mg/dL), and serum lactate was elevated at 3.6 mmol/L. Electrolyte analysis revealed mild hyponatremia (131 mmol/L) and hypokalemia (3.2 mmol/L). Arterial blood gas testing was consistent with a metabolic acidosis with partial respiratory compensation (Table 1).

**Table 1:** Initial laboratory values on admission Laboratory values were obtained at the time of admission before initiation of resuscitative or antimicrobial therapy. The findings reflect systemic inflammation, early sepsis, and metabolic derangement associated with bowel perforation. Reference ranges are based on standard institutional laboratory parameters and may vary slightly by laboratory.

Test	Patient value	Reference range
White blood cell count	17.4 × 10⁹/L	4-11 × 10⁹/L
Neutrophils	88%	40%-75%
Hemoglobin	12.8 g/dL	12-16 g/dL (female)
Hematocrit	37.2%	36%-46% (female)
Platelet count	263 × 10⁹/L	150-400 × 10⁹/L
Serum sodium	131 mmol/L	135-145 mmol/L
Serum potassium	3.2 mmol/L	3.5-5.0 mmol/L
Serum creatinine	2.1 mg/dL	0.6-1.1 mg/dL (female)
Blood urea nitrogen	36 mg/dL	7-20 mg/dL
C-reactive protein	86 mg/L	<5 mg/L
Serum lactate	3.6 mmol/L	0.5-2.2 mmol/L
Arterial pH	7.29	7.35-7.45
Bicarbonate	17 mmol/L	22-29 mmol/L
Base excess	-6.2 mmol/L	-2 to +2 mmol/L

**Figure 1:**
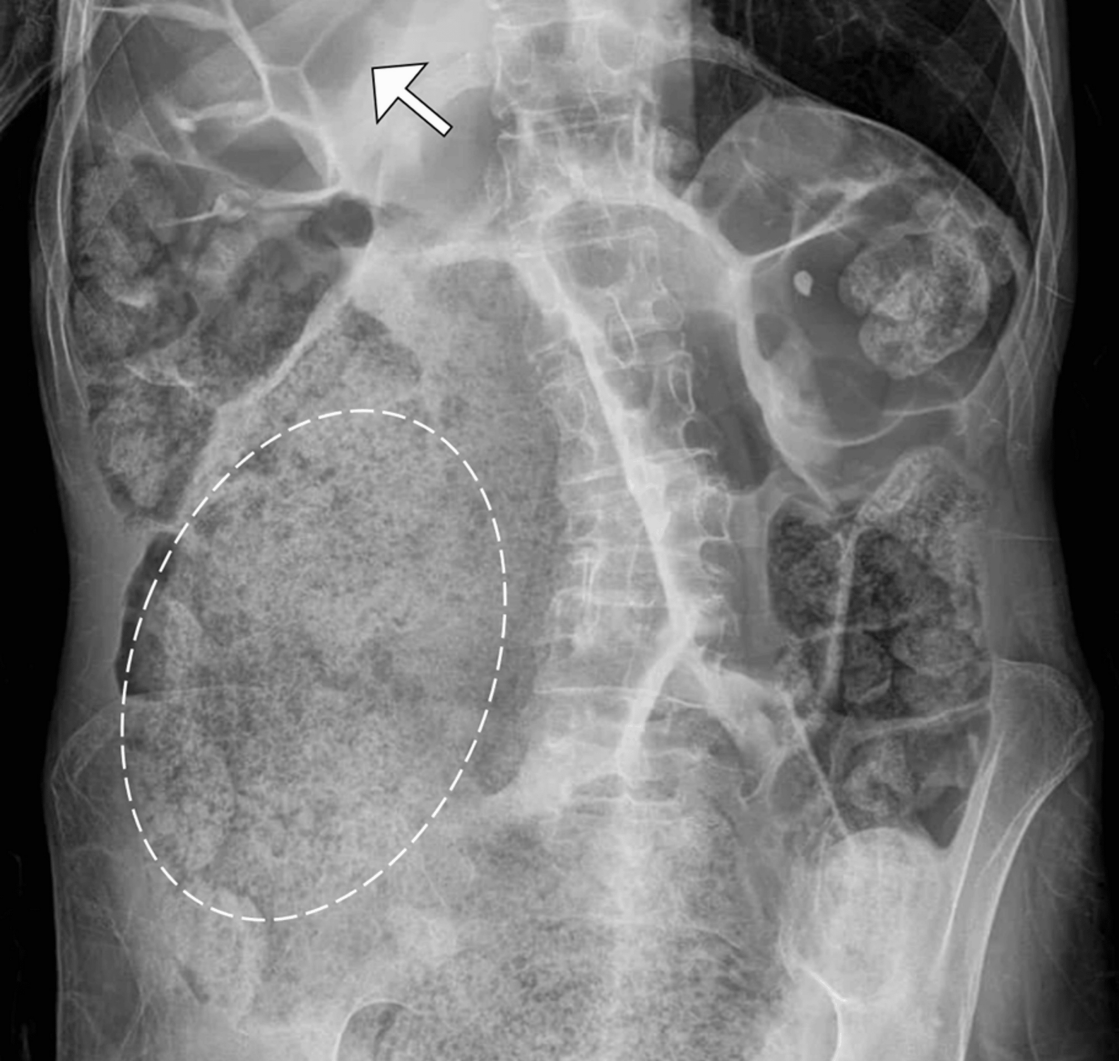
Abdominal radiograph demonstrating fecaloma and colonic distension Supine abdominal X-ray showing extensive fecal material within the colon (encircled), with marked colonic dilatation (arrow). No evidence of free intraperitoneal air is observed on this view.

**Figure 2:**
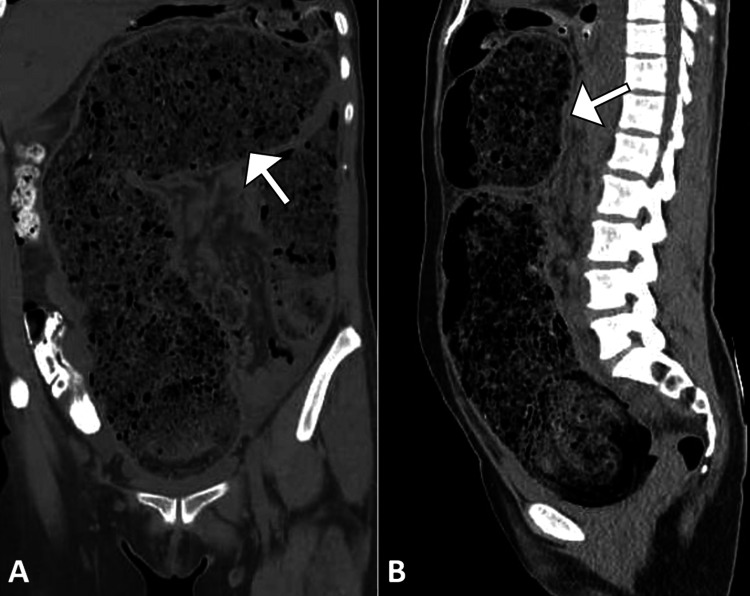
Coronal and sagittal CT images demonstrating fecal impaction and colonic dilatation (A) Coronal and (B) sagittal views from a contrast-enhanced abdominal CT scan showing large-volume fecal material impacted throughout the colon, with associated luminal dilatation (arrow). The findings are consistent with severe fecal loading and impending perforation. CT: computed tomography

Initial evaluation with a plain abdominal radiograph revealed marked colonic dilatation along with a sizable fecal mass occupying the colon (Figure 1). A subsequent contrast-enhanced computed tomography (CT) scan of the abdomen and pelvis identified a large, compacted fecal mass within the distal sigmoid colon, consistent with a fecaloma. Associated findings included focal thinning of the colonic wall, surrounding inflammatory fat stranding, and the presence of free intraperitoneal air and fluid, findings indicative of sigmoid colon perforation (Figure 2). No radiologic evidence of colorectal malignancy, volvulus, or ischemic changes in other bowel segments was observed.

Based on the clinical presentation and radiologic findings, the differential diagnoses considered included stercoral colitis complicated by perforation due to fecaloma, perforated sigmoid diverticulitis, colorectal malignancy with perforation, and ischemic colitis leading to secondary bowel rupture. The lack of radiologic evidence of diverticular disease, obstructing tumor, or mesenteric vascular insufficiency, combined with the presence of severe fecal impaction, favored a diagnosis of colonic perforation attributable to a fecaloma.

The patient was immediately managed with aggressive intravenous fluid resuscitation, initiation of broad-spectrum antimicrobial therapy with piperacillin-tazobactam, and placement of a nasogastric tube for gastric decompression. Following hemodynamic stabilization, she was taken urgently to the operating theater for an exploratory laparotomy. Intraoperative findings included a 1.8 cm perforation located along the antimesenteric aspect of the sigmoid colon, directly adjacent to a densely impacted fecaloma. Localized purulent contamination with fibrinous deposits was noted, without evidence of diffuse fecal peritonitis. Surgical management consisted of a Hartmann’s procedure, involving resection of the diseased sigmoid segment and creation of an end colostomy. The fecaloma was manually evacuated intraoperatively. Thorough peritoneal irrigation was performed, and a pelvic drain was placed before closure.

Following surgery, the patient was transferred to the surgical intensive care unit for close postoperative surveillance. She required short-term vasopressor therapy but subsequently maintained stable hemodynamics. Microbiological cultures isolated *Escherichia coli*, which was sensitive to the empirically initiated antimicrobial therapy. Nutritional support was progressively advanced, and the patient began mobilizing with assistance by the fifth postoperative day. The colostomy became functional on postoperative day 4, accompanied by the return of normal bowel sounds. The pelvic drain was removed on day 6 after demonstrating minimal output. Her recovery progressed without complication, and she was discharged in good condition on postoperative day 11 with arrangements for outpatient surgical follow-up, stoma care instruction, and continuation of oral osmotic laxatives.

At the one-month follow-up visit, the patient remained asymptomatic and demonstrated complete clinical recovery. She reported adherence to dietary modifications, including increased fiber intake and adequate daily hydration, along with consistent use of prescribed laxatives. The chronic nature of her constipation was discussed and acknowledged. Consideration for elective colostomy reversal was postponed pending full recovery and nutritional optimization. Ongoing follow-up with both the surgical and gastroenterology services has been maintained.

## Discussion

Colonic perforation resulting from a fecaloma represents a rare but severe manifestation within the spectrum of stercoral disease, typically developing as a consequence of prolonged constipation and sustained intraluminal pressure leading to bowel wall injury. The clinical scenario observed in our elderly patient with a long history of constipation, who developed sigmoid colon perforation, is consistent with patterns reported in previous studies. Published series indicate that chronic constipation is present in approximately 69%-81% of affected patients, with reported median ages ranging from the late fifth to early sixth decade of life [1-3]. The sigmoid colon has been identified as the most frequently involved segment, accounting for nearly half of cases, with perforations commonly occurring along the antimesenteric border due to relative vascular vulnerability and elevated intraluminal pressure from impacted fecal material [2].

This case highlights the critical role of early contrast-enhanced computed tomography in establishing the diagnosis and guiding timely surgical intervention. In our patient, CT imaging demonstrated characteristic features including a fecaloma, pericolic inflammatory changes, focal thinning of the colonic wall, and pneumoperitoneum, facilitating rapid operative decision-making. These findings are consistent with prior reports documenting diagnostic accuracies of approximately 90%-95% for CT in stercoral disease, with colonic dilatation exceeding 6 cm, wall thickening greater than 3 mm, pericolic fat stranding, and extraluminal air recognized as key radiologic indicators [2-4].

The underlying mechanism of stercoral perforation is believed to involve a sequence of pressure-induced ischemia, mucosal ulceration, transmural necrosis, and eventual rupture of the bowel wall. This pathophysiological process has been well described in earlier case series, in which histopathological analysis revealed deep ulceration and full-thickness necrosis at sites of fecaloma contact [5,6]. Although histologic examination was not required in the present case, intraoperative findings were consistent with these established observations.

Several predisposing factors have been associated with stercoral perforation, including opioid use, limited mobility, advanced age, institutionalization, and comorbid conditions such as diabetes mellitus and chronic renal disease. In this patient, underlying chronic kidney disease may have contributed to impaired gastrointestinal motility, in keeping with epidemiological data demonstrating a high burden of comorbid illness among affected individuals [7-10].

Surgical intervention remains the cornerstone of management for fecaloma-associated colonic perforation. Hartmann’s procedure is most commonly employed and was successfully performed in this case, involving prompt laparotomy, removal of the fecaloma, resection of the perforated segment, extensive peritoneal lavage, and formation of an end colostomy. Despite advances in perioperative care, stercoral perforation continues to carry a substantial mortality risk, with reported rates ranging from approximately 17% to as high as 34%-57% in cases complicated by delayed diagnosis or advanced sepsis [6,7]. The favorable outcome observed in our patient likely reflects early recognition, rapid imaging, and timely surgical management, consistent with recent reports suggesting improved survival with heightened clinical awareness [5-7]. Nevertheless, stercoral perforation remains an underdiagnosed entity, and this case further emphasizes the importance of early CT evaluation in elderly patients with constipation presenting with acute abdominal symptoms, potentially averting progression to severe sepsis or septic shock.

## Conclusions

This case highlights that long-standing, poorly managed constipation can escalate into a life-threatening surgical emergency. The formation of a fecaloma and the resulting sigmoid colon perforation in this patient emphasize the need to take chronic bowel complaints seriously, especially in older adults with reduced physiological reserve. Early recognition of subtle clinical changes and prompt use of diagnostic imaging are crucial for timely intervention, potentially transforming a critical presentation into a favorable outcome. Preventive measures, including consistent adherence to bowel management plans, early involvement of gastroenterology specialists, and education of high-risk populations, are key strategies to reduce the risk of fecaloma formation and its severe complications.
